# Artificial reefs facilitate tropical fish at their range edge

**DOI:** 10.1038/s42003-019-0398-2

**Published:** 2019-05-06

**Authors:** Avery B. Paxton, Charles H. Peterson, J. Christopher Taylor, Alyssa M. Adler, Emily A. Pickering, Brian R. Silliman

**Affiliations:** 1 Institute of Marine Sciences, University of North Carolina at Chapel Hill, 3431 Arendell Street, Morehead City, NC 28557 USA; 2 Department of Biology, University of North Carolina at Chapel Hill, 120 South Road, Chapel Hill, NC 27599 USA; 3 National Ocean Service, National Centers for Coastal Ocean Science, National Oceanic and Atmospheric Administration, 101 Pivers Island Road, Beaufort, NC 28516 USA; 4 Nicholas School of the Environment, Duke University Marine Lab, 135 Duke Marine Lab Road, Beaufort, NC 28516 USA; 5 Present Address: Nicholas School of the Environment, Duke University Marine Lab, 135 Duke Marine Lab Road, Beaufort, NC 28516 USA

**Keywords:** Urban ecology, Community ecology

## Abstract

Spatial planning increasingly incorporates theoretical predictions that artificial habitats assist species movement at or beyond range edges, yet evidence for this is uncommon. We conducted surveys of highly mobile fauna (fishes) on artificial habitats (reefs) on the southeastern USA continental shelf to test whether, in comparison to natural reefs, artificial reefs enhance local abundance and biomass of fishes at their poleward range margins. Here, we show that while temperate fishes were more abundant on natural reefs, tropical, and subtropical fishes exhibited higher abundances and biomasses on deep (25–35 m) artificial reefs. Further analyses reveal that this effect depended on feeding guilds because planktivorous and piscivorous but not herbivorous fishes were more abundant on artificial reefs. This is potentially due to heightened prey availability on and structural complexity of artificial reefs. Our findings demonstrate that artificial habitats can facilitate highly mobile species at range edges and suggest these habitats assist poleward species movement.

## Abstract (web summary)

Avery Paxton et al. test the effects of artificial reefs on abundance and biomass of fish on the southeastern coast of the USA. They report that deep artificial reefs are associated with high numbers and biomasses of tropical and subtropical fishes, likely caused by an increase in prey availability on artificial reefs.

## Introduction

Global rates of urbanization are increasing. For example, urbanized landscapes are projected to triple in area by 2030, relative to 2000^1^. These unprecedented levels of urbanization reflect changing human settlement patterns where percentages of people living in urban settings increased from 30% in 1950 to 54% in 2014, with expectations of reaching 67% by 2050^2^. Coastlines experience particularly concentrated development since one-third of humans reside within 100 km of the coast^3^. Urbanization, however, is no longer restricted to terrestrial and coastal environments and now extends beneath the surface of the ocean, a phenomenon known as marine urbanization or ocean sprawl^4,5^. Unintended consequences of installing artificial habitats can drive ecological changes ranging from biodiversity degradation^6^ and biotic homogenization^7^ to community-level shifts^8^ and the spread of invasive species^9^. With a forecasted increase in the number of artificial habitats, minimizing negative ecological effects and also maximizing positive outcomes associated with artificial habitats is pressing.

Increases in number of artificial habitats are now happening at the same time as widely documented shifts in species ranges^10,11^. With changing climate conditions, for example, terrestrial species generally move towards higher latitudes or higher elevations, and marine species generally move poleward and deeper^12^. Species anticipated to exhibit range shifts require habitat corridors or stepping stones, to move across habitat-limited areas or areas with degraded habitats as they track suitable environmental conditions^13,14^. Indeed, recent literature has called for strategic establishment of additional stepping stones, such as restored habitats or supplemented habitats, to facilitate species movement^15,16^. This strategy, however, is based primarily on theory^13,15,17,18^, and the effectiveness of creating habitat corridors targeting species at range edges is unknown. Tests of whether strategically placed, artificial habitats may facilitate species at their range edges, providing a potentially positive benefit, require a study system, where widespread installation of artificial structures has occurred for a long time and where some species occur near their range edges.

**Fig. 1:**
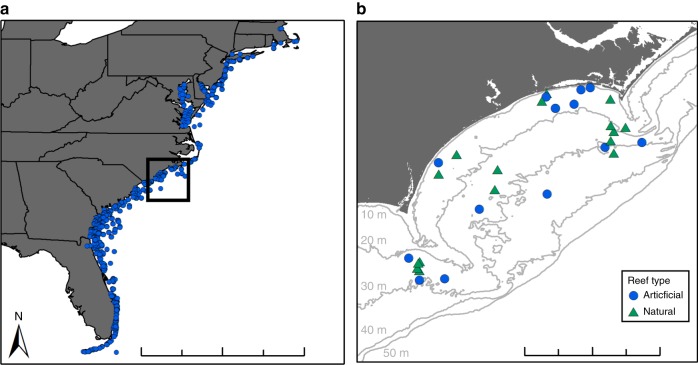
**a** Artificial reefs established along the eastern USA. Black square indicates location of (**b**) 30 warm-temperate reefs surveyed on the inner continental shelf. Gray lines indicate bathymetric contours, beginning with 10 m deep closest to shore. Scale divisions are (**a**) every 250 km and (**b**) every 25 km. Data in panel **a** are from MarineCadastre.gov (BOEM and NOAA. MarineCadastre.gov. Artificial Reefs. Accessed 2018 from marinecadastre.gov/data)

Given that marine species range shifts occur faster than shifts in terrestrial species’ ranges^19^ and that some marine fishes have been documented to be moving poleward and deeper^11^, we searched for a model marine system containing artificial habitats frequented by reef fishes. A primary habitat enhancement method in marine systems is to create new habitat by intentionally sinking human-made, artificial structures to form artificial reefs. Artificial reefs are widespread globally^20,21^, and in the USA, for example, many states maintain active artificial reef programs (Fig. 1a). Although there is support for the idea that artificial reefs can enhance fish production^22^, it is unknown whether they can enhance local abundance and biomass of fishes at their range edges. As such, we used artificial reefs to test whether artificial habitats can facilitate mobile species (fishes) at their range edges.

Theory and previous research suggest that artificial reefs could enhance local abundance and biomass of fishes at their range margins. For instance, artificial reefs form connectivity corridors that facilitate movement of benthic invertebrates and macroalgae, including several non-native species, among habitats^9,23,24^. These human-made reefs can also support a higher proportion of transient fish species than neighboring natural reefs^25^. Moreover, offshore renewable energy infrastructure serves as stepping stones for fishes moving among habitats and is hypothesized to form important corridors for fishes at biogeographic boundaries that may be near their range edges^26^. Whether artificial structures can facilitate movement of fishes and other marine fauna at their range edges and potentially poleward and deeper is unknown. Such a function could be a positive effect of installation of artificial habitats.

Here, we explored whether artificial reefs may facilitate fishes at their range edges. We tested whether and how artificial reefs and natural reefs provide habitat for fishes with different climate ranges: tropical, subtropical, and temperate by conducting diving surveys of 30 warm-temperate reefs on the continental shelf of North Carolina (NC), USA. These reefs are located near a biogeographic transition zone^27,28^ and host a diversity of tropical, subtropical, and temperate fishes^29,30^. Fishes on these warm-temperate reefs that are tropical species, as well as many subtropical species, are considered close to their range edges. We specifically asked: do abundance and biomass of tropical, subtropical, and temperate fishes differ on artificial and natural reefs; do reef variables, including reef depth, water temperature, and structural complexity predict these differences; what potential mechanisms drive observed differences; and how does this affect a subset of fishes observed poleward of their documented range edges? Answering these questions is important for guiding installation and regulation of artificial habitats in the context of global change. We show that temperate fishes were more abundant on natural reefs but that tropical and subtropical fishes exhibited higher abundances and biomasses on deep (25–35 m) artificial reefs. Additional analyses revealed that this effect depended on feeding guilds and likely also on heightened prey availability on or increased structural complexity of artificial reefs. Our findings demonstrate that artificial habitats can facilitate highly mobile species at range edges and suggest these habitats assist poleward species movement.

## Results

### Fish abundance and biomass on reefs

**Fig. 2:**
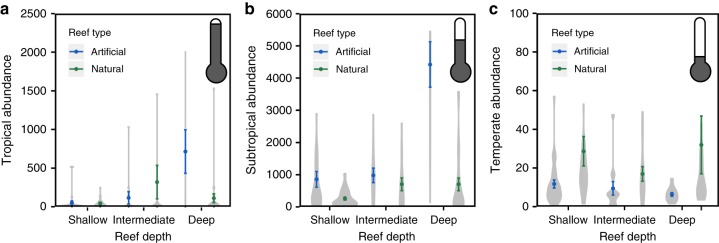
Abundance (±1 SE) of fishes (per 120 m^2^) on artificial reefs versus natural reefs by fish climate range: (**a**) tropical, (**b**) subtropical, (**c**) temperate. Reef depth zones are: shallow: 5–18 m, intermediate: 18–25 m, deep: 25–35 m. Shaded areas of the violin plots are proportional to the number of observations. Points represent mean observed abundance (±1 SE). GLM results appear in Supplementary Table 3

Visual surveys of fish communities across 14 artificial reefs and 16 rocky reefs (Fig. 1b; Supplementary Table 2), revealed that relative abundance and biomass of fishes characterized by tropical, subtropical, and temperate ranges differed on artificial and natural reefs (Fig. 2; Supplementary Table 3; Supplementary Fig. 1). These surveys also demonstrate that fish abundance and biomass differ with environmental factors, such as reef depth (e.g., average bottom depth of reef), water temperature, and reef complexity, which were measured simultaneously along the same visual transects where fish were counted and identified. For tropical fishes, both reef depth and reef type (natural versus artificial; GLM reef type *P* = 0.04, depth *P* *<* 0.0001; reef type × depth *P* = 0.02) influenced their abundance. Specifically, tropical fish abundance was similar on natural and artificial reefs shallower than 25 m. On deeper reefs, however, numbers of tropical fishes on artificial reefs exceeded those on natural reefs (Fig. 2a). For subtropical fishes, abundance was influenced not only by reef type and depth, similar to for tropical fishes, but also reef complexity (GLM reef type *P* < 0.0001, depth *P* < 0.0001, reef complexity *P* = 0.02, reef complexity^2^ *P* *<* 0.01). Shallow-depth and intermediate-depth artificial and natural reefs supported similar numbers of subtropical fishes, whereas deep artificial reefs supported more subtropical fishes than natural reefs (Fig. 2b). Reef complexity exhibited a unimodal relationship with subtropical fish abundance, where abundance increased until reaching intermediate levels of reef complexity, after which fish abundance decreased. Temperate fishes occurred in higher numbers on natural reefs than artificial reefs, as well as on colder reefs (Fig. 2c; Supplementary Table 3; GLM reef type *P* < 0.0001, depth *P* = 0.08, temperature *P* < 0.0001). Fish biomass exhibited a similar pattern to fish abundance with tropical fishes and subtropical fishes displaying higher biomass on deep, artificial reefs (Supplementary Fig. 1).

### Potential mechanisms

**Fig. 3:**
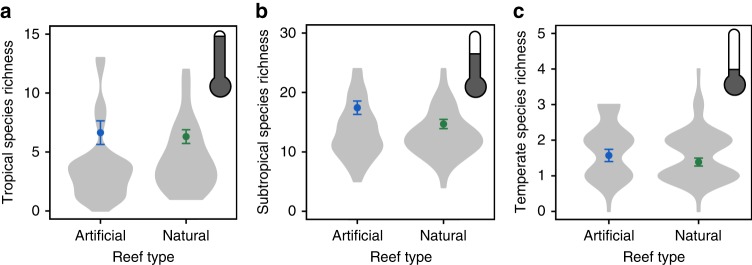
Species richness (per 120 m^2^) of fishes on deep (18–25 m) artificial reefs versus natural reefs for (**a**) tropical, (**b**) subtropical, and (**c**) temperate climate ranges. Shaded areas of the violin plots are proportional to the number of observations. Points represent mean observed species richness (±1 SE)

**Fig. 4:**
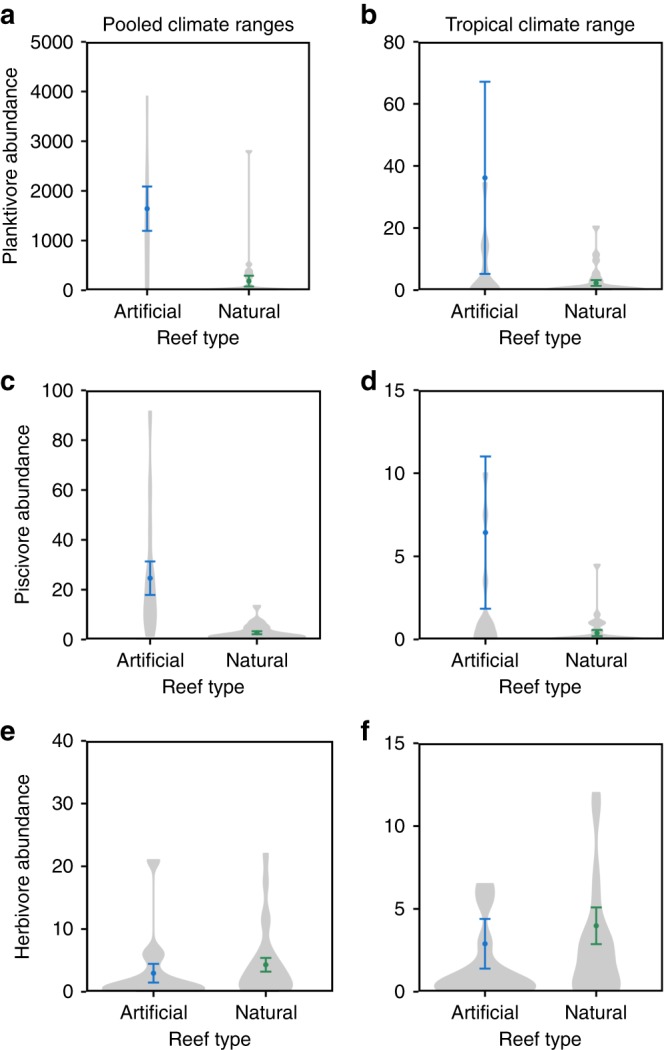
Abundance (per 120 m^2^) of fish trophic groups on deep (18–25 m) artificial reefs versus natural reefs. (**a**) All climate ranges (tropical, subtropical, temperate) of planktivorous fishes, (**b**) tropical planktivorous fishes, (**c**) all climate ranges of piscivorous fishes, (**d**) tropical piscivorous fishes, (**e**) all climate ranges of herbivorous fishes, and (**f**) tropical herbivorous fishes. Shaded areas of the violin plots are proportional to the number of observations. Points represent mean observed abundance (±1 SE). GLM model results appear in Supplementary Table 4

To test potential mechanisms for differing numbers of tropical, subtropical, and temperate fishes on deep artificial versus deep natural reefs, we examined species richness. Artificial and natural reefs had similar numbers of tropical and temperate species (Fig. 3; GLM reef type *P* > 0.05), indicating that reefs differed primarily in the abundance and biomass of species rather than the number of species present. Subtropical fishes, however, exhibited higher species richness on artificial than natural reefs (Fig. 3b; GLM reef type *P* = 0.04). Next, we tested whether abundances of fish feeding guilds (e.g., herbivore, planktivore, etc.) differed by reef type. We found that, when pooling fishes from all climate ranges, deep artificial reefs hosted more planktivorous fishes (Fig. 4a; Supplementary Table 4; GLM reef type *P* = 0.02) and piscivorous fishes (Fig. 4c; Supplementary Table 4; GLM reef type *P* < 0.0001) than deep, natural reefs. This pattern of elevated abundance on artificial versus natural reefs remained consistent for planktivores and piscivores with strictly tropical climate ranges (Fig. 4b, c; Supplementary Table 4). In contrast, deep, natural reefs hosted equivalent numbers of herbivorous fishes, which were mainly tropical fishes, as did artificial reefs (Fig. 4e, f; Supplementary Table 4; GLM reef type *P* > 0.05). Numbers of generalist feeders, such as invertivores (Supplementary Fig. 2; Supplementary Table 4; GLM reef type *P* < 0.0001) and omnivores (Supplementary Fig. 2; Supplementary Table 4; GLM reef type *P* = 0.02), although higher overall on artificial reefs, displayed largely species-specific differences in abundance by reef type, driven by primary prey resources. For example, omnivores that consume primarily macroalgae, occurred in higher abundances on natural reefs, whereas omnivores that consume primarily zooplankton occurred in higher abundances on artificial reefs.

### Fishes poleward of documented ranges

We also examined relative abundance and prey preferences of tropical, as well as several subtropical, fishes whose normal range limits were documented in Fishbase^31^ as south of the studied reefs. These fishes represent a small subset of tropical fishes encountered on the reefs since they were observed rarely and in low abundances. These fishes may also be currently at the margins of their ranges. When we compared abundances of these fishes at range edges, we discovered that those fishes documented to consume zooplankton or nekton occurred in higher numbers on artificial reefs. Fishes that consume macroalgae exhibited higher abundances on natural reefs (Supplementary Fig. 3; Supplementary Table 5). For example, fish species that exhibited higher abundances on artificial reefs included planktivores (*Chromis cyanea* (blue chromis) and *Thalassoma bifasciatum* (bluehead wrasse) and piscivores (*Mycteroperca interstitialis* (yellowmouth grouper), as well as more generalist feeders. Of the generalist feeders, several consume plankton and nekton as part of their diet or are associated with predatory fishes. For example, *Anisotremus surinamensis* (black margate), although an invertivore, consumes zooplankton and small fishes, and *Bodianus rufus* (Spanish hogfish) consumes parasites off of large, predatory fishes. In contrast, fish species that exhibited higher abundance on natural reefs included herbivores (*Stegastes variabilis* (cocoa damselfsih) and *Sparisoma atomarium* (green blotch parrotfish), as well as generalist feeders. One generalist feeder with higher numbers on natural reefs, *Abudefduf taurus* (night sergeant), also consumes macroalgae as part of its diet. Two-sample *t*-tests indicated that these species-specific differences in abundance by reef type were not significant.

## Discussion

We provide evidence that fishes with different climate ranges exhibited distinct preferences for artificial versus natural reefs. Temperate fishes occurred in higher abundance and biomass on natural, rocky reefs, whereas subtropical fishes and tropical fishes at deep depths (25–35 m) resided in higher numbers and biomass on artificial reefs. Further analysis revealed that this effect depended on feeding guilds because planktivorous and piscivorous fishes but not herbivorous fishes were more abundant on artificial reefs. This effect, which persisted among tropical fishes at their poleward range edges, is potentially due to heightened prey availability on or increased structural complexity of artificial reefs. These findings demonstrate that artificial reefs enhance local abundance and biomass of fishes at their range edges. Additionally, these results suggest that artificial reefs facilitate poleward movement of tropical fishes by providing suitable habitat corridors, where temperature regimes occur within thermal tolerances of the species and where reef trophic structure matches prey preferences of the species.

Elevated numbers of tropical fishes on artificial reefs compared to natural reefs likely depends on prey preference. Our observation that planktivorous and piscivorous fishes occurred in higher numbers on artificial reefs has been consistently documented in other comparative studies of artificial versus natural reefs^32,33^. These planktivores and piscivores may occur in such high numbers on artificial reefs because of heightened prey availability. Zooplankton consumed by planktivorous fishes, as well as planktivorous fishes consumed by piscivorous fishes, occur in consistent spatial patterns around artificial structures^34^. Zooplanktivory has also been documented to be a key process around artificial reefs^35^. In contrast, macroalgae cover is higher on natural than artificial reefs in the study area, which could explain why herbivorous fishes did not occur in higher numbers on artificial reefs.

Increased structural complexity of artificial reefs compared to natural reefs may also explain why artificial reefs enhance local abundance of species at range edges. A previous study of the same 30 reefs surveyed here demonstrated that artificial reefs are on average three times more structurally complex than nearby natural reefs^30^. With increased structural complexity, artificial reefs may provide refuge from predators, facilitating survival of tropical fishes, especially those recruiting towards the margins of their ranges. By extension, natural reefs may offer less pronounced protection from predators, potentially preventing establishment of high abundances of tropical species. While tropical fish abundance and biomass were elevated on artificial reefs, our study also revealed that natural reefs provide habitat for similar numbers of tropical species as artificial reefs. This pattern is distinct from previous observations where both abundance and richness of tropical fishes were elevated at range edges for corals^36^, as well as for macroalgae, other invertebrates, and fishes^37^. This contrasting pattern suggests that elevated abundance and biomass of species at their range edge in our study may be isolated to species near their range edges but already present at these reefs in low abundances. This would make sense, as species range shifts are gradual processes and because our study provides a snapshot of fish communities during our sampling events. We have not documented species range shifts but rather that tropical species have higher abundance and biomass on deep artificial reefs than natural reefs. Future research should be conducted to track fish species over time to observe the range shift process and test whether they actively occur on these reefs.

Select tropical fishes, as well as several subtropical species, occurred poleward of their generally characterized ranges. These fishes have likely been present on the surveyed reefs for years in low or variable abundances, avoiding detection and subsequent reporting, especially as annual fluctuations in winter temperatures control fish communities^29^. Because of their uncommon occurrences as far north as the reefs we studied, however, we classified these fishes as a subset of the larger group of tropical fishes located near their range edges. While statistical tests comparing abundances of this subset of species by reef type were not significant, this is not surprising given that these tests were on a subset of very rarely observed tropical and subtropical fishes. This non-significant finding does not detract from our overall finding that tropical fishes, which are likely to be at their range edges on the sampled warm-temperate reefs, have elevated abundance and biomass on artificial reefs. Instead, the subset of fishes observed poleward of their documented ranges provide a more qualitative case study for understanding how fishes moving poleward while tracking suitable water temperatures^11^ may utilize artificial and natural reefs. Our results suggest that prey preferences may drive fish habitat choice in waters outside of their normal geographic ranges but within their thermal tolerances. We posit that when expanding to waters beyond their normal ranges, fishes that consume zooplankton and nekton would preferentially utilize artificial habitats. In contrast, fishes that consume macroalgae would preferentially occupy natural reef habitats which host elevated coverage of macroalgae compared to artificial habitats (personal observation, A. Paxton), even though most of these herbivorous fishes are tropical species. Because artificial reefs form optimal habitat for planktivorous and piscivorous tropical fishes, artificial reefs have the potential to facilitate poleward movement of tropical fishes. Facilitation of tropicalization is plausible, especially as artificial reefs facilitate a similar process, the introduction of invasive species, such as some macroalgae and invertebrates^23,24^. Whereas subtropical and tropical fish are not invasive species, the habitat-related mechanisms of invasion and tropicalization are likely comparable.

While we provide evidence that artificial reefs facilitate species at range edges, our findings add to a growing body of evidence that artificial reefs may act as beneficial artificial habitats by facilitating movement of fishes and other marine fauna poleward and deeper. If artificial reefs are confirmed to facilitate poleward movement of marine fauna, then strategic deployment of artificial reefs and other artificial habitats across swaths of the coastal ocean may create corridors permitting animal movement among areas with degraded habitats or that are otherwise habitat limited. In the global context of artificial habitat installation, our findings on artificial reefs demonstrate that some types of artificial habitats can enhance local abundance and biomass of species at range edges, potentially conferring added benefits of habitat supplementation.

## Methods

### Survey sites

We conducted SCUBA-diver surveys of 30 temperate reefs off the coast of NC along the southeastern USA continental shelf (Fig. 1b; Supplementary Table 1). Fourteen of the 30 sites are artificial reefs, including shipwrecks, as well as concrete pipes and ships, purposely sunk as part of the NC Artificial Reef Program^38^. Sixteen are natural reefs, representing flat-type to ledge-type morphologies. Reefs span 1.3° of latitude, ranging from 33.4 N to 34.7 N. Twenty-three of these reefs occur within Onslow Bay, NC, whereas the remaining seven sites lie farther south in northeastern Long Bay, NC within an area designated for potential offshore wind energy development, but all are considered temperate reefs. Reefs in Onslow Bay were selected a priori based on a design that was stratified by water depth, which is correlated with distance from shore. Sites in Long Bay were selected from side-scan sonar and multibeam bathymetry datasets acquired during a seafloor mapping cruise in June 2013^39^. Sites were sampled seasonally during 2013–2015 (Supplementary Table 1). Seasons were classified as: winter (January–March), spring (April–June), summer (July–September), and fall (October–December). Most sites were sampled during each season, but several were sampled fewer times because of rough sea conditions. At each site, two 30-m long transects were established along prominent reef features. If the reef lacked a prominent feature, we used a list of randomly generated compass headings to select the transect direction. Transect location at each site varied among seasons. Diver surveys to quantify fishes, reef depth, and water temperature were conducted along each transect.

### Fish community

To quantify fish abundance, divers sampled along a 30 m × 4 m (120 m^2^) belt transect^40–42^, while recording species and abundance of all fishes present throughout the water column. Each belt-transect included both conspicuous and cryptic categories of reef fishes that were identified to the lowest taxonomic level possible. Fish length was estimated visually to the nearest cm. Biomass was calculated with the length–weight power function as

$$W = aL^{\mathrm{{b}}}$$

where *L* is length (cm) recorded on the fish transect, and *W* is weight (g). When a school of fishes spanned multiple sizes, *L* was calculated as the midpoint of the recorded size range. Species-specific morphometric values for *a* and *b* were obtained from Fishbase^31^. For species that were identifiable only to the family level, the average morphometric values for other known species in the family present on the reefs were used. Weight was converted to kg. Fish climate ranges were assigned as temperate, subtropical, or tropical using published classifications from Fishbase^31^ and Whitfield et al.^29^, which designated species as ‘tropical’ if their northern distribution limit occurred at the northern Atlantic coast of FL, ‘subtropical’ if their range extended poleward to NC, and ‘temperate’ if their range encompassed the northeastern US. For fishes identified to the family level, the predominant climate range of other species in that family also present on the reef was assigned. Species whose northernmost distributional latitude in Fishbase^31^ fell south of studied reefs were also identified, but we caution that latitudinal ranges reported in Fishbase do not always include rare sightings at higher latitudes. When two belt transects were conducted at a reef during a single sampling season, the fish abundances from each transect were each averaged as a single replicate to characterize abundance; this was also conducted for biomass and richness.

### Structural complexity

As per methods developed by Dustan et al.^43^ and implemented by Paxton et al.^30^, we collected measurements of the contour of each reef using an Onset HOBO U20 Titanium Water Level Logger (U20-001-02-Ti) containing a pressure-transducer that records pressure at 1 Hz, from which bottom elevations were inferred. A diver swam over the reef with the logger suspended from a line and positioned as close to the substrate as possible. If benthic organisms, such as sponges, coral, and dense meadows of macroalgae, rose above the substrate preventing us from positioning the logger close to the substrate, then we moved the logger above these habitat-forming animals and plants to avoid damaging them and to account for them in our complexity measurement. The logger was moved at ~ 10 cm/s over the length of each 30-m transect. The logger was raised 1 m above and rapidly lowered back down to the substrate surface in a spike motion five times at the start of each transect, three times every 5 m thereafter, and five times at the end of each transect. Since the logger records continuously, these spikes were used to identify each transect within the data record and convert sample time to distance along transects. During post-dive processing, distance calibration spikes were removed from each file, and raw pressures recorded by the pressure-transducer were converted from units of psi to m, assuming an atmospheric pressure of 1 atm. If the diver swim-speed differed from the target rate of ~10 cm per second, then the actual swim speed was computed from the transect length and time between calibration spikes and used to determine distance along the 30-m transect. Digital reef rugosity (DRR)^43^ was calculated as the standard deviation of depths along each transect (m) and was used as our metric for reef complexity, such that higher values of reef rugosity indicate a more structurally complex reef.

### Reef depth

We used pressure measurements collected by the same Onset HOBO U20 Titanium Water Level Logger (U20-001-02-Ti) that we used to measure reef complexity to also calculate mean depth along each transect. We used these mean depths to categorically classify reef depth as: shallow: 5–18 m, intermediate: 18–25 m, deep: 25–35 m for visualizations but retained reef depth as a continuous variable for statistical analyses.

### Water temperature

We measured temperature on each transect using the same Onset HOBO U20 Titanium Water Level Logger (U20-001-02-Ti) that we used to measure reef complexity and reef depth. The logger recorded temperature every second over the duration of each transect. Because temperature often changes with season and because we were examining fishes whose climate ranges (e.g., tropical, subtropical, temperate) are associated with water temperatures, we used water temperature in subsequent statistical analyses instead of seasons.

### Statistical analyses

Statistical analyses were conducted in R version 3.3.2^44^. We used generalized linear models (GLMs) to test whether reef type (artificial, natural), depth, water temperature, and structural complexity affected abundance and biomass of fishes by individual climate ranges. For fish abundance and biomass, we constructed GLMs with a negative-binomial error distribution and a log-link function within the ‘MASS’ package^45^. Fish abundance values from each reef were originally integers, but because we conducted two transects per reef during each sampling season, we later averaged abundances from replicate transects to avoid pseudoreplication. Averaging resulted in non-integer abundances, so prior to performing GLMs, we rounded the mean abundance data to the nearest integer, since we did not encounter fractions of fish and since the negative-binomial distribution requires integers. Similarly, we also rounded mean biomass data to the nearest integer. One species of temperate fish, *Menidia menidia* (Atlantic silverside), were present in large schools on only several surveys, so these fishes were removed from abundance and biomass analyses.

Models were run individually for abundance and biomass of fishes belonging to each of the three climate ranges. For each response variable (e.g., abundance for a specific climate range), the most complex GLM was fit first and then compared to candidate models of reduced complexity until reaching the most parsimonious model via a simultaneous comparison of candidate models. The most complex models regressed fish community metrics against reef type, depth, water temperature, and both a linear term for structural complexity and a squared term for structural complexity. We included both the linear and squared terms for structural complexity because of previously documented unimodal relationships between fish abundance and reef complexity^30^. We also included water temperature as a predictor variable, which helps account for effects of seasonality.

Model selection from among our most complex and more parsimonious candidate models was conducted using Akaike information criterion (AIC) values based on minimum AIC. If two models had close AIC values (e.g., within two units of each other), then the more parsimonious model was selected. We conducted graphical and analytical assessments of fit to compare the predicted values from the model to the observed values. For the graphical assessment of fit, we plotted the estimated probability distribution with the observed fish community metric values superimposed. For analytical assessments of fit, we calculated *P*-values where the observed value of fish community metrics was treated as the test statistic and the predicted probability distribution was treated as the null model.

To investigate potential mechanisms of patterns observed in the abundance and biomass models, we constructed several additional models and fit them as explained above. First, we modeled the relationship between reef type and fish species richness by constructing GLMs with a Poisson distribution for each climate range. Second, to investigate differences in abundance of functional groups by reef type, we constructed GLMs with a negative-binomial error distribution and a log-link function. Third, for fishes observed on the reefs whose normal range limits were documented in Fishbase^31^ as south of the studied reefs, we calculated their average abundances on artificial and natural reefs. We visualized whether these fishes were more abundant on artificial or natural reefs by plotting the mean abundance of each selected fish by reef type. Because many fishes exhibited zero-abundance or low-abundance on one or both reef types, we log-transformed the abundance values as follows:

$${\mathrm{log}}({\mathrm{{abundance}}} + 0.01)$$

to better visualize plotted data. Two-sample *t*-tests quantified whether the mean abundances of these fishes statistically differed by reef type.

### Reporting summary

Further information on experimental design is available in the Nature Research Reporting Summary linked to this article.

## Supplementary information

Supplementary material

Reporting Summary

## Data Availability

The datasets generated during and/or analyzed during the current study are available from the corresponding author on reasonable request.
