# Supplementary material for: Artificial reefs facilitate tropical fish at their range edge
Source: Commun Biol. 2019 May 6;2:168. doi: 10.1038/s42003-019-0398-2 (PMC6502939; doi:10.1038/s42003-019-0398-2)
Supplement: Supplementary file 1 — Supplementary material [file 42003_2019_398_MOESM1_ESM.pdf]

Supplementary Information

Supplementary Figures

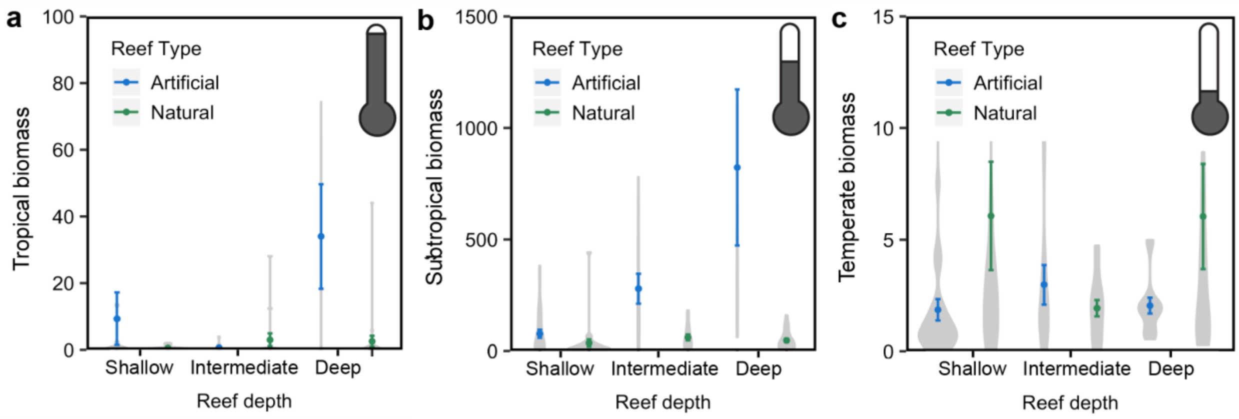

Supplementary Figure 1: Biomass of fishes (kg per 120 m^2) on artificial reefs versus natural reefs by fish climate range: a) tropical, b) subtropical, c) temperate. Reef depth zones are: shallow: 5-18 m, intermediate: 18-25 m, deep: 25-35 m. Shaded areas of the violin plots are proportional to the number of observations. Points represent mean observed biomass (± 1 SE). GLM results appear in Supplementary Table 3.

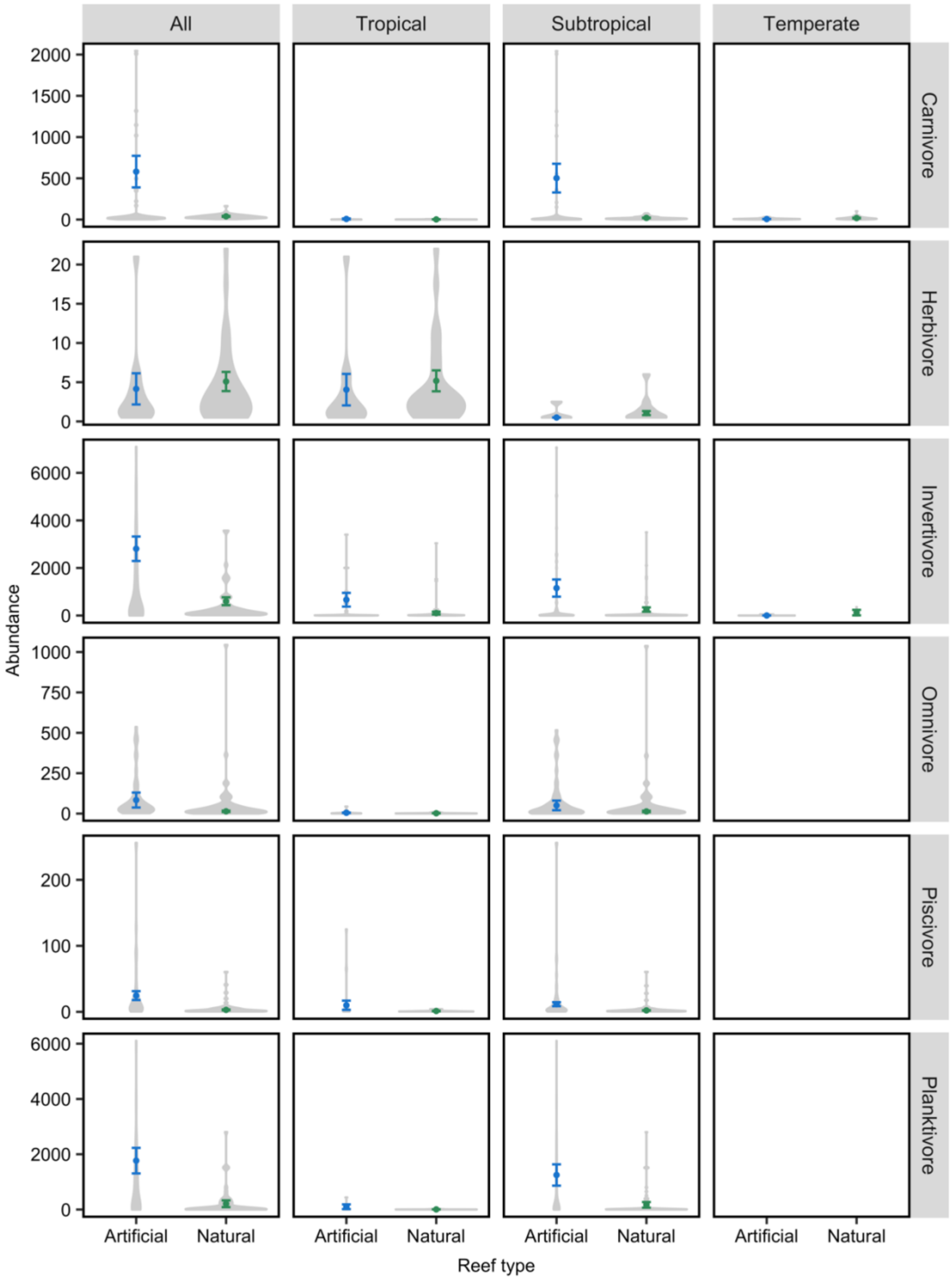

Supplementary Figure 2: Abundance (± 1 SE) of fishes (per 120 m^2) on deep (18-25 m) artificial reefs versus natural reefs by fish climate range and fish trophic group. GLM results appear in Supplementary Table 4.

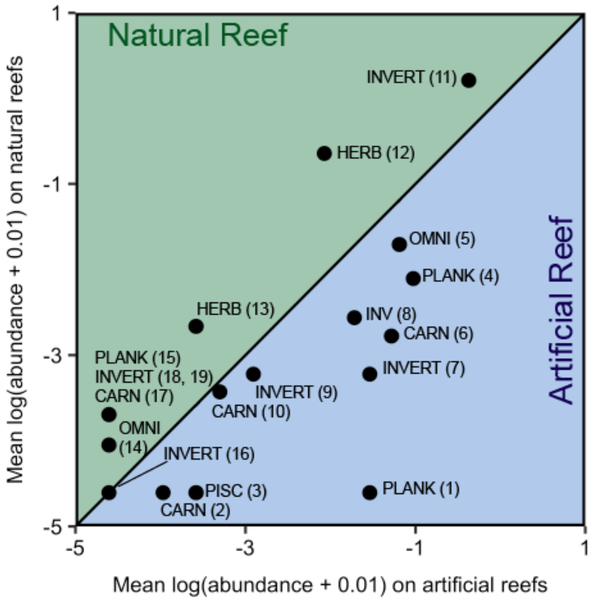

Supplementary Figure 3: Mean abundance (log (abundance + 0.01) (per 120 m^2)) of tropical and subtropical fishes at their range edges. Each fish is indicated by a point. Text labels indicate trophic group (PLANK = planktivore, CARN = carnivore, HERB = herbivore, OMNI = omnivore, INVERT = invertivores). Fishes falling in the blue region of the graph, below the 1:1 line, occur in higher numbers on artificial reefs. Fishes in the green region, above the 1:1 line, display higher abundance on natural reefs. Metadata for these fishes are displayed in Supplementary Table 6, referenced by the number is parentheses.

Supplementary Tables

Supplementary Table 1: Descriptions of thirty reefs surveyed. Coordinates are provided to the nearest tenth of a degree for latitude (Lat) and longitude (Lon). Mean environmental variables include depth (Dep), reef complexity measured as digital reef rugosity (DRR), water temperature (Tem), and sediment standard deviation (Sed; natural reefs only). Date indicates month and year (month/year) of replicate transects. Coordinates of a previously unknown wreck are purposely excluded.

**Reef name**	**Reef type**	**Structure**	**Lat (dd)**	**Lon (dd)**	**Dep (m)**	**DRR (m)**	**Tem (ºC)**	**Date**
Theodore Parker (AR-315)	Artificial	Ship	34.7	-76.7	10.2	0.5	24.3	7/8/2013, 10/29/13, 7/29/14, 10/2/14, 10/20/14
Atlantic Beach Bridge (AR-320)	Artificial	Concrete	34.7	-76.8	15.2	0.8	23.0	10/31/2013, 7/29/14, 9/29/14, 10/20/14, 5/19/15
Concrete Pipes, 2007 (AR-342)	Artificial	Concrete	34.6	-77.0	16.1	0.4	24.2	9/6/2013, 6/3/14, 8/20/14, 10/21/14, 5/19/15
USS Indra (AR-330)	Artificial	Ship	34.6	-76.9	15.0	1.1	24.0	6/9/2014, 8/21/14, 5/18/15
Titan (AR-345)	Artificial	Ship	34.5	-77.0	16.6	0.8	23.0	7/18/2013, 10/28/13, 6/2/14, 10/2/14, 10/21/14, 5/19/15
Concrete Pipes, 2006 (AR-345)	Artificial	Concrete	34.5	-77.0	18.7	0.2	22.9	7/18/2013, 10/28/13, 6/2/14, 10/2/14, 10/21/14, 5/19/15
Yard Oiler FS-26 (AR-300)	Artificial	Ship	34.3	-76.4	26.4	1.4	24.6	6/17/14
Spar (AR-305)	Artificial	Ship	34.3	-76.6	28.3	1.4	25.1	8/30/2013, 6/16/14, 10/10/14
Alexander Ramsey	Artificial	Ship	34.2	-77.8	12.0	2.3	24.7	9/19/2013, 7/1/14, 9/5/14, 11/13/14
Cassimir	Artificial	Ship	34.0	-77.0	32.6	0.8	25.4	9/4/2014, 11/5/14
John D. Gill	Artificial	Ship	33.9	-77.5	25.1	0.7	25.0	9/24/2013, 6/24/14, 8/7/14, 11/13/14
Raritan	Artificial	Ship	33.5	-77.9	21.5	1.7	23.3	9/25/2013, 6/25/14, 9/11/14, 12/18/14
City of Houston	Artificial	Ship	33.4	-77.7	28.0	0.4	27.7	9/25/2013, 6/25/14, 9/11/14
Unknown Wreck	Artificial	Ship	-----	-----	29.0	0.5	24.1	12/4/2013, 6/25/14, 9/11/14
Keypost Rock	Natural	Ledge	34.6	-77.0	15.0	0.4	25.0	10/24/14
Barge Rock	Natural	Ledge	34.6	-76.6	16.1	0.6	24.8	8/19/2013, 7/30/14, 7/29/14, 10/20/14

Station Rock	Natural	Pavement & rubble	34.6	-77.1	15.6	0.3	23.4	9/6/2013, 10/30/13, 6/2/14, 8/20/14, 10/21/14, 5/19/15
Northwest Reef	Natural	Ledge	34.4	-76.6	21.4	0.7	23.8	8/12/2013, 6/17/14, 10/10/14, 10/20/14, 5/18/15
Southwest of Knuckle Buoy	Natural	Pavement & rubble	34.4	-76.5	14.2	0.2	23.2	8/20/2013, 10/28/13, 10/20/14, 5/18/15
10 Fathom	Natural	Ledge	34.4	-76.6	20.9	0.6	23.7	8/20/2013, 10/29/13, 6/17/14, 8/22/14, 10/20/14, 5/18/15
West Rock	Natural	Pavement & rubble	34.3	-76.6	24.9	0.3	25.2	9/4/2013, 6/16/14, 10/10/14, 5/27/15
210 Rock	Natural	Pavement & rubble	34.2	-76.6	30.2	0.2	25.2	9/4/2013, 6/16/14, 10/10/14
Dallas Rocks	Natural	Pavement & rubble	34.2	-77.6	16.5	0.3	24.7	9/20/2013, 7/1/14, 9/4/14, 11/5/14
200 / 200 Ledge	Natural	Ledge	34.1	-77.4	25.1	0.3	23.8	9/20/2013, 11/5/14
5 Mile Ledge	Natural	Ledge	34.1	-77.8	15.8	0.4	24.7	9/19/2013, 7/1/14, 9/5/14, 11/13/14
23 Mile Ledge	Natural	Ledge	34.0	-77.4	28.7	0.4	25.5	6/24/2014, 9/5/14
Hammerhead Ledge	Natural	Pavement & rubble	33.5	-77.9	25.4	0.1	21.8	12/13/2013, 6/30/14, 9/11/14, 12/19/14
Thumb Ledge	Natural	Pavement & rubble	33.5	-77.9	26.5	0.2	26.5	9/25/2013, 6/25/14, 9/11/14
Lightning Bolt Ledge	Natural	Pavement & rubble	33.5	-77.9	28.5	0.2	21.8	12/13/2013, 6/25/14, 9/11/14, 12/19/14
Bumpy Ledge	Natural	Pavement & rubble	33.5	-77.9	29.2	0.1	21.0	12/13/2013, 6/25/14, 12/15/14

Supplementary Table 2: Fish, shark, and turtle species list from 226 fish belt-transects conducted on warm-temperate reefs of the NC continental shelf. Abundance values indicate the total number of each species observed across the 226 transects on artificial reefs (AR), natural reefs (NR), and in total (Total). Bold indicates members of the federally-managed snapper-grouper complex.

**Family**	**Genus**	**Species**	**Common name**	**Climate range**	**Functional group**	**Habitat zone**	**AR**	**NR**	**Total**
Acanthuridae	*Acanthurus*	*chirurgus*	Doctorfish	Subtropical	Herbivore	Demersal	8	23	31
Acanthuridae	*Acanthurus*	coeruleus	Blue tang surgeonfish	Tropical	Herbivore	Demersal	1	0	1
Anguillidae	*Anguilla*	*rostrata*	American eel	Subtropical	Invertivore	Demersal	0	2	2
Apogonidae	*Apogon*	*pseudomaculatus*	Twospot cardinalfish	Tropical	Invertivore	Demersal	10	72	82
Apogonidae	*Apogon*	*planifrons*	Pale cardinalfish	Tropical	Invertivore	Demersal	0	2	2
Atherinopsidae	*Menidia*	*menidia*	Silversides	Temperate	Planktivore	Pelagic	14255	10320	24575
**Balistidae**	***Balistes***	***capriscus***	**Grey triggerfish**	**Subtropical**	**Invertivore**	**Demersal**	**9**	**51**	**60**
Batrachoididae	*Opsanus*	*tau*	Oyster toadfish	Subtropical	Benthic Carnivore	Demersal	17	31	48
Belonidae	*Ablennes*	*hians*	Flat needlefish	Subtropical	Piscivore	Demersal	0	3	3
Blenniidae	*Parablennius*	*marmoreus*	Seaweed blenny	Subtropical	Planktivore	Demersal	177	271	448
Blenniidae	*Blenniidae*	sp.	Unknown blenny	Tropical	Omnivore	Demersal	88	22	110
Blenniidae	*Hypleurochilus*	*geminatus*	Crested blenny	Subtropical	Omnivore	Demersal	18	0	18
Blenniidae	*Ophioblennius*	*macclurei*	Redlip blenny	Tropical	Omnivore	Demersal	0	1	1
Carangidae	*Decapterus*	*macarellus*	Mackerel scad	Subtropical	Planktivore	Pelagic	36866	19636	56502
Carangidae	*Decapterus*	*punctatus*	Round scad	Subtropical	Planktivore	Pelagic	24256	5883	30139
Carangidae	*Selar*	*crumenophthalmus*	Bigeye scad	Subtropical	Carnivore	Pelagic	9260	52	9312
Carangidae	*Decapterus*	sp.	Scad species	Subtropical	Planktivore	Pelagic	1100	0	1100
**Carangidae**	***Seriola***	***dumerili***	**Greater amberjack**	**Subtropical**	**Piscivore**	**Pelagic**	**640**	**114**	**754**
Carangidae	*Caranx*	*crysos*	Blue runner	Subtropical	Benthic Carnivore	Pelagic	32	225	257
Carangidae	*Carangoides*	*bartholomaei*	Yellow jack	Subtropical	Piscivore	Pelagic	165	72	237
**Carangidae**	***Seriola***	***rivoliana***	**Almaco jack**	**Subtropical**	**Piscivore**	**Pelagic**	**84**	**13**	**97**

**Carangidae**	***Caranx***	***ruber***	**Bar jack**	**Subtropical**	**Omnivore**	**Pelagic**	**55**	**7**	**62**
Carcharhinidae	*Carcharhinus*	*plumbeus*	Sandbar shark	Subtropical	Carnivore	Shark	1	0	1
Chaetodontidae	*Chaetodon*	*ocellatus*	Spotfin butterflyfish	Tropical	Omnivore	Demersal	8	15	23
Chaetodontidae	*Chaetodon*	*sedentarius*	Reef butterflyfish	Subtropical	Omnivore	Demersal	3	7	10
Cheloniidae	*Caretta*	*caretta*	Loggerhead turtle	Subtropical	Herbivore	Turtle	0	1	1
Cheloniidae	*Cheloniidae*	sp.	Unknown turtle	Subtropical	Herbivore	Turtle	0	1	1
Dasyatidae	*Dasyatis*	*americana*	Southern stingray	Subtropical	Benthic Carnivore	Demersal	8	0	8
Diodontidae	*Chilomycterus*	*schoepfi*	Striped burrfish	Tropical	Invertivore	Demersal	3	3	6
Diodontidae	*Chilomycterus*	*antennatus*	Bridled burrfish	Tropical	Invertivore	Demersal	2	0	2
Echeneidae	*Remora*	*remora*	Remora	Subtropical	Carnivore	Demersal	5	3	8
**Ephippidae**	***Chaetodipterus***	***faber***	**Atlantic spadefish**	**Subtropical**	**Invertivore**	**Pelagic**	**494**	**248**	**742**
Gobiidae	*Coryphopterus*	*eidolon*	Pallid goby	Tropical	Herbivore	Demersal	25	47	72
Gobiidae	*Coryphopterus*	*glaucofraenum*	Bridled goby	Tropical	Omnivore	Demersal	25	34	59
Gobiidae	*Gobiidae*	sp.	Unknown goby	Tropical	Omnivore	Demersal	26	24	50
Gobiidae	*Gnatholepsis*	*thompsoni*	Goldspot goby	Tropical	Omnivore	Demersal	0	2	2
Grammatidae	*Gramma*	*loreto*	Fairy basslet	Tropical	Invertivore	Demersal	0	2	2
Ginglymostom-atidae	*Ginglymostoma*	*cirratum*	Nurse shark	Subtropical	Benthic Carnivore	Shark	1	0	1
**Haemulidae**	***Haemulon***	***aurolineatum***	**Tomtate**	**Subtropical**	**Invertivore**	**Pelagic**	**92563**	**25676**	**118239**
Haemulidae	*Haemulidae*	sp.	Unknown juvenile grunt	Tropical	Invertivore	Demersal	23308	14461	37769
Haemulidae	*Orthopristis*	*chrysoptera*	Pigfish	Temperate	Invertivore	Demersal	137	1312	1449
**Haemulidae**	***Haemulon***	***plumierii***	**White grunt**	**Subtropical**	**Invertivore**	**Demersal**	**124**	**464**	**588**
Haemulidae	*Anisotremus*	*surinamensis*	Black margate	Subtropical	Invertivore	Demersal	19	9	28
**Haemulidae**	***Haemulon***	***album***	**White margate**	**Tropical**	**Invertivore**	**Demersal**	**0**	**1**	**1**
Kyphosidae	*Kyphosus*	*sectatrix*	Bermuda chub	Subtropical	Omnivore	Demersal	1	2	3
Labridae	*Halichoeres*	*bivittatus*	Slippery dick	Tropical	Invertivore	Demersal	683	2005	2688
Labridae	*Halichoeres*	*caudalis*	Painted wrasse	Subtropical	Invertivore	Demersal	18	151	169
Labridae	*Tautoga*	*onitis*	Tautog	Temperate	Benthic Carnivore	Demersal	26	70	96
Labridae	*Thalassoma*	*bifasciatum*	Bluehead wrasse	Tropical	Planktivore	Demersal	39	15	54

Labridae	*Bodianus*	*rufus*	Spanish hogfish	Tropical	Invertivore	Demersal	23	4	27
Labridae	*Halichoeres*	*radiatus*	Puddingwife	Tropical	Invertivore	Demersal	11	10	21
Labridae	*Labridae*	sp.	Unknown wrasse	Tropical	Invertivore	Demersal	15	0	15
**Labridae**	***Lachnolaimus***	***maximus***	**Hogfish**	**Subtropical**	**Invertivore**	**Demersal**	**5**	**0**	**5**
Lotidae	*Brosme*	*brosme*	Cusk	Temperate	Invertivore	Demersal	1	0	1
**Lutjanidae**	***Rhomboplites***	***aurorubens***	**Vermilion snapper**	**Subtropical**	**Benthic Carnivore**	**Demersal**	**18571**	**193**	**18764**
**Lutjanidae**	***Lutjanus***	***campechanus***	**Red snapper**	**Subtropical**	**Benthic Carnivore**	**Demersal**	**8**	**22**	**30**
**Lutjanidae**	***Lutjanus***	***mahogoni***	**Mahogany snapper**	**Subtropical**	**Benthic Carnivore**	**Demersal**	**9**	**17**	**26**
**Lutjanidae**	***Lutjanus***	***synagris***	**Lane snapper**	**Subtropical**	**Benthic Carnivore**	**Demersal**	**4**	**2**	**6**
**Lutjanidae**	***Lutjanus***	***griseus***	**Gray snapper**	**Subtropical**	**Benthic Carnivore**	**Demersal**	**5**	**0**	**5**
**Lutjanidae**	***Ocyurus***	***chrysurus***	**Yellowtail snapper**	**Subtropical**	**Invertivore**	**Demersal**	**0**	**1**	**1**
Monacanthidae	*Stephanolepis*	*hispidus*	Planehead filefish	Subtropical	Benthic Carnivore	Demersal	28	24	52
Mullidae	*Mullus*	*auratus*	Red goatfish	Subtropical	Benthic Carnivore	Demersal	105	2	107
Mullidae	*Pseudupeneus*	*maculatus*	Spotted goatfish	Subtropical	Benthic Carnivore	Demersal	7	45	52
Mullidae	*Upeneus*	*parvus*	Dwarf goatfish	Tropical	Benthic Carnivore	Demersal	34	8	42
Mullidae	*Mulloidichthys*	*martinicus*	Yellow goatfish	Subtropical	Benthic Carnivore	Demersal	30	7	37
Muraenidae	*Muraena*	*retifera*	Reticulate moray	Subtropical	Benthic Carnivore	Demersal	2	10	12
Muraenidae	*Gymnothorax*	*miliaris*	Goldentail moray	Subtropical	Benthic Carnivore	Demersal	1	3	4
Muraenidae	*Muraenidae*	sp.	Unknown moray eel	Subtropical	Piscivore	Demersal	0	1	1
Odontaspididae	*Carcharias*	*taurus*	Sand tiger shark	Subtropical	Piscivore	Pelagic	48	1	49
Ophichthidae	*Myrichthys*	*ocellatus*	Goldspotted eel	Tropical	Benthic Carnivore	Demersal	2	0	2
Osteichthyes	*Osteichthyes*	sp.	Unknown fish species	Subtropical	Omnivore	Demersal	3	12	15
Ostraciidae	*Acanthostracion*	*polygonius*	Honeycomb cowfish	Tropical	Invertivore	Demersal	0	1	1
Ostraciidae	*Acanthostracion*	*quadricornis*	Scrawled cowfish	Subtropical	Invertivore	Demersal	1	0	1
Ostraciidae	*Lactophrys*	*triqueter*	Smooth trunkfish	Subtropical	Invertivore	Demersal	1	0	1
Paralichthyidae	*Paralichthys*	*albigutta*	Gulf flounder	Subtropical	Benthic Carnivore	Demersal	11	25	36
Paralichthyidae	*Paralichthys*	*dentatus*	Summer flounder	Temperate	Benthic Carnivore	Demersal	13	8	21
Paralichthyidae	*Paralichthys*	*lethostigma*	Southern flounder	Subtropical	Benthic Carnivore	Demersal	4	1	5

Phycidae	*Urophycis*	*earllii*	Carolina hake	Subtropical	Benthic Carnivore	Demersal	5	17	22
Pomacanthidae	*Holacanthus*	*bermudensis*	Blue angelfish	Subtropical	Invertivore	Demersal	65	66	131
Pomacanthidae	*Holacanthus*	*ciliaris*	Queen angelfish	Subtropical	Invertivore	Demersal	5	4	9
Pomacanthidae	*Pomacanthidae*	sp.	Unknown juvenile angelfish	Subtropical	Invertivore	Demersal	0	3	3
Pomacentridae	*Chromis*	*scotti*	Purple reeffish	Tropical	Planktivore	Demersal	962	60	1022
Pomacentridae	*Pomacentridae*	sp.	Unknown juvenile damselfish	Tropical	Herbivore	Demersal	26	85	111
Pomacentridae	*Stegastes*	*leucostictus*	Beaugregory damselfish	Tropical	Omnivore	Demersal	78	26	104
Pomacentridae	*Stegastes*	*variabilis*	Cocoa damselfish	Tropical	Herbivore	Demersal	13	69	82
Pomacentridae	*Stegastes*	*partitus*	Bicolor damselfish	Tropical	Herbivore	Demersal	29	42	71
Pomacentridae	*Chromis*	*cyanea*	Blue chromis	Tropical	Planktivore	Demersal	23	0	23
Pomacentridae	*Stegastes*	*diencaeus*	Longfin damselfish	Tropical	Omnivore	Demersal	9	0	9
Pomacentridae	*Stegastes*	*adustus*	Dusky damselfish	Tropical	Herbivore	Demersal	3	0	3
Pomacentridae	*Abudefduf*	*saxatilis*	Sergeant-major	Tropical	Omnivore	Demersal	1	1	2
Pomacentridae	*Chromis*	*enchrysura*	Yellowtail reeffish	Tropical	Planktivore	Demersal	0	2	2
Pomacentridae	*Abudefduf*	*taurus*	Night sergeant	Subtropical	Omnivore	Demersal	0	1	1
Ptereleotridae	*Ptereleotris*	*calliura*	Blue dartfish	Tropical	Planktivore	Demersal	0	27	27
Rachycentridae	*Rachycentron*	*canadum*	Cobia	Subtropical	Carnivore	Pelagic	1	0	1
Rajidae	*Dipturus*	*laevis*	Barndoor skate	Temperate	Carnivore	Demersal	0	1	1
Scaridae	*Sparisoma*	*atomarium*	Greenblotch parrotfish	Tropical	Herbivore	Demersal	2	7	9
Scaridae	*Scarus*	*iseri*	Striped parrotfish	Subtropical	Herbivore	Demersal	0	3	3
Sciaenidae	*Pareques*	*umbrosus*	Cubbyu	Subtropical	Benthic Carnivore	Demersal	617	669	1286
Sciaenidae	*Pareques*	*acuminatus*	High-hat	Tropical	Benthic Carnivore	Demersal	3	3	6
Scombridae	*Scomberomorus*	*maculatus*	Spanish mackerel	Subtropical	Piscivore	Pelagic	592	2	594
Scombridae	*Scomberomorus*	*cavalla*	King mackerel	Tropical	Piscivore	Pelagic	250	0	250
Scombridae	*Euthynnus*	*alletteratus*	Little tunny	Tropical	Piscivore	Pelagic	146	8	154
Scorpaenidae	*Pterois*	*volitans*	Lionfish	Tropical	Piscivore	Demersal	35	12	47
Scorpaenidae	*Scorpaena*	*plumieri*	Spotted scorpionfish	Subtropical	Benthic Carnivore	Demersal	1	2	3
**Serranidae**	***Centropristis***	***striata***	**Black sea bass**	**Temperate**	**Benthic Carnivore**	**Demersal**	**919**	**2116**	**3035**

Serranidae	*Serranus*	*subligarius*	Belted sandfish	Subtropical	Invertivore	Demersal	494	703	1197
**Serranidae**	***Mycteroperca***	***microlepis***	**Gag**	**Subtropical**	**Benthic Carnivore**	**Demersal**	**190**	**185**	**375**
**Serranidae**	***Centropristis***	***ocyurus***	**Bank sea bass**	**Subtropical**	**Benthic Carnivore**	**Demersal**	**45**	**197**	**242**
**Serranidae**	***Mycteroperca***	***phenax***	**Scamp**	**Subtropical**	**Piscivore**	**Demersal**	**91**	**106**	**197**
Serranidae	*Rypticus*	*maculatus*	White spotted soapfish	Subtropical	Benthic Carnivore	Demersal	60	55	115
Serranidae	*Diplectrum*	*formosum*	Sand perch	Subtropical	Benthic Carnivore	Demersal	5	34	39
**Serranidae**	***Epinephelus***	***guttatus***	**Red hind**	**Tropical**	**Invertivore**	**Demersal**	**2**	**0**	**2**
Serranidae	*Hypoplectrus*	*puella*	Barred hamlet	Tropical	Invertivore	Demersal	0	2	2
Serranidae	*Serranus*	*baldwini*	Lantern bass	Tropical	Benthic Carnivore	Demersal	0	2	2
Serranidae	*Serranus*	*phoebe*	Tattler bass	Subtropical	Invertivore	Demersal	0	2	2
Serranidae	*Serranus*	*tigrinus*	Harlequin bass	Tropical	Invertivore	Demersal	0	2	2
Serranidae	*Liopropoma*	*eukrines*	Wrasse bass	Subtropical	Benthic Carnivore	Demersal	0	1	1
**Serranidae**	***Mycteroperca***	***interstitialis***	**Yellowmouth grouper**	**Subtropical**	**Piscivore**	**Demersal**	**1**	**0**	**1**
Serranidae	*Rypticus*	*saponaceus*	Greater soapfish	Tropical	Benthic Carnivore	Demersal	1	0	1
Sparidae	*Diplodus*	*holbrookii*	Spottail pinfish	Subtropical	Omnivore	Demersal	10053	6513	16566
**Sparidae**	***Stenotomus***	***chrysops***	**Scup**	**Subtropical**	**Benthic Carnivore**	**Demersal**	**533**	**474**	**1007**
Sparidae	*Archosargus*	*probatocephalus*	Sheepshead	Subtropical	Omnivore	Demersal	372	135	507
Sparidae	*Lagodon*	*rhomboides*	Pinfish	Subtropical	Omnivore	Demersal	192	83	275
Sparidae	*Calamus*	*penna*	Sheepshead porgy	Tropical	Invertivore	Demersal	76	161	237
**Sparidae**	***Stenotomus***	***caprinus***	**Longspine porgy**	**Subtropical**	**Benthic Carnivore**	**Demersal**	**56**	**171**	**227**
Sparidae	*Calamus*	*proridens*	Littlehead porgy	Subtropical	Invertivore	Demersal	2	122	124
**Sparidae**	***Calamus***	***calamus***	**Saucereye porgy**	**Subtropical**	**Invertivore**	**Demersal**	**46**	**67**	**113**
**Sparidae**	***Calamus***	***nodosus***	**Knobbed porgy**	**Subtropical**	**Invertivore**	**Demersal**	**2**	**26**	**28**
**Sparidae**	***Calamus***	***bajonado***	**Jolthead porgy**	**Subtropical**	**Invertivore**	**Demersal**	**5**	**10**	**15**
**Sparidae**	***Sparidae***	**sp.**	**Unknown porgy**	**Tropical**	**Invertivore**	**Demersal**	**74**	**6**	**80**
Sparidae	*Archosargus*	*rhomboidalis*	Sea bream	Subtropical	Omnivore	Demersal	4	0	4
**Sparidae**	***Pagrus***	***pagrus***	**Red porgy**	**Subtropical**	**Invertivore**	**Demersal**	**3**	**0**	**3**
Sphyraenidae	*Sphyraena*	*guachancho*	Guaguanche	Subtropical	Piscivore	Pelagic	590	200	790

Sphyraenidae	*Sphyraena*	*barracuda*	Barracuda	Subtropical	Piscivore	Pelagic	73	7	80
Sphyraenidae	*Sphyraena*	*borealis*	Northern sennet	Subtropical	Piscivore	Pelagic	0	1	1
Synodontidae	*Synodus*	*foetens*	Inshore lizardfish	Tropical	Piscivore	Demersal	1	3	4
Tetraodontidae	*Canthigaster*	*rostrata*	Sharpnose puffer	Tropical	Omnivore	Demersal	33	23	56
Tetraodontidae	*Sphoeroides*	*spengleri*	Bandtail puffer	Subtropical	Benthic Carnivore	Demersal	10	11	21

Supplementary Table 3: Generalized linear model (GLM) results for fish abundance and biomass by fish climate range (tropical, subtropical, temperate). Environmental variables include reef type (artificial versus natural, average reef depth (m), reef complexity (m), squared reef complexity (m), and water temperature (^{o}C). Bold values indicate significance. Model results displayed here are from the best models that we evaluated. GLMs used negative-binomial distributions.

Fish climate range	Metric	Predictor variable	df	Deviance	Residual df	Residual deviance	*P*
Tropical	Abundance	Null			123	203.0	
		**Reef type**	1	4.3	122	198.7	**0.04**
		**Depth**	1	35.6	121	163.2	**<0.0001**
		**Reef type X Depth**	1	5.7	120	157.5	**0.02**
Subtropical	Abundance	Null			123	241.8	
		**Reef type**	1	41.9	122	199.9	**<0.0001**
		**Depth**	1	42.1	121	157.7	**<0.0001**
		**Complexity**	1	5.1	120	152.6	**0.02**
		**Complexity^2**	1	8.7	119	143.9	**<0.01**
Temperate	Abundance	Null			123	204.2	
		**Reef type**	1	36.7	122	167.4	**<0.0001**
		Depth	1	3.1	121	164.4	0.08
		**Temperature**	1	29.1	120	135.3	**<0.0001**
Tropical	Biomass	Null			123	92.3	
		**Reef type**	1	12.3	122	80.0	**<0.001**
		**Depth**	1	6.9	121	73.1	**<0.01**
Subtropical	Biomass	Null			123	283.6	
		**Reef type**	1	85.5	122	198.1	**<0.0001**
		**Depth**	1	32.7	121	165.5	**<0.0001**
		**Reef type X Depth**	1	20.1	120	145.4	**<0.0001**
Temperate	Biomass	Null			123	146.1	
		**Reef type**	1	12.9	122	132.6	**<0.001**
		**Temperature**	1	6.4	121	126.1	**0.01**

Supplementary Table 4: Generalized linear model (GLM) results for fish abundance by functional group on deep (18-25 m) reefs. Environmental variables include reef type (artificial versus natural). Bold values indicate significance with significance. Model results displayed here are from the best models that we evaluated. GLMs used negative-binomial distributions.

**Fish functional group**	**Fish climate range**	**Predictor variable**	**df**	**Deviance**	**Residual df**	**Residual deviance**	***P***
Planktivores	All	Null			39	58.3	
		**Reef type**	1	8.3	38	50.0	**<0.001**
Planktivores	Tropical	Null			39	33.2	
		**Reef type**	1	7.1	38	26.1	**<0.01**
Piscivores	All	Null			39	93.6	
		**Reef type**	1	51.3	38	42.3	**<0.0001**
Piscivores	Tropical	Null			39	38.5	
		**Reef type**	1	13.4	38	25.1	**<0.001**
Herbivores	All	Null			39	42.7	
		Reef type	1	0.6	38	42.1	0.4
Herbivores	Tropical	Null			39	39.4	
		Reef type	1	0.3	38	39.1	0.6
Omnivores	All	Null			39	63.0	
		**Reef type**	1	15.3	38	47.7	**<0.0001**
Omnivores	Tropical	Null			39	46.3	
		**Reef type**	1	6.1	38	40.2	**0.01**
Invertivores	All	Null			39	65.8	
		**Reef type**	1	18.4	38	47.4	**<0.0001**
Invertivores	Tropical	Null			39	63.5	
		**Reef type**	1	10.8	38	52.8	**<0.01**
Carnivores	All	Null			39	112.9	
		**Reef type**	1	67.5	38	45.3	**<0.0001**
Carnivores	Tropical	Null			39	11.0	
		Reef type	1	0.9	38	10.1	0.3

Supplementary Table 5: Fishes not commonly reported as far north as surveyed reefs. Each species includes its scientific name, common name, climate range, and northern latitude (N_lat; [1], as well as mean count ± standard error on artificial (Artificial_Mean; N = 55) and natural (Natural_Mean; N = 63). Reef preference indicates whether fishes exclusively occurred on artificial (AR_exclusive) or natural (NR_exclusive) reefs or whether they generally resided on artificial (AR) or natural (NR) reefs. Prey consumed by each species is provided. Northern latitudes represent the commonly documented and preferred latitudes from Fishbase [1]. Some may seem counterintuitive. For example, *Mulloidichtys martinicus* has a northern latitude of 32°N and is subtropical species while *Thalassoma bifasciatum* has a northern latitude of 32°N but is classified as tropical. These classifications are based on preferred and commonly documented ranges, as well as climate ranges assigned in Whitfield et al. 2014 [2].

**#**	**Scientific name**	**Common name**	**Climate range**	**N lat**	**Artificial (mean ± se)**	**Natural (mean ± se)**	**Reef_ preference**	**Prey**
1	*Chromis cyanea*	Blue chromis	Tropical	32	0.21 ± 0.20	0.00 ± 0.00	AR_ exclusive	Planktivore
2	*Myrichthys ocellatus*	Goldspotted eel	Tropical	25	0.02 ± 0.02	0.00 ± 0.00	AR_ exclusive	Benthic Carnivore
3	*Mycteroperca interstitialis*	Yellowmouth grouper	Subtropical	33	0.01 ± 0.01	0.00 ± 0.00	AR_ exclusive	Piscivore
4	*Thalassoma bifasciatum*	Bluehead wrasse	Tropical	32	0.35 ± 0.24	0.12 ± 0.09	AR	Planktivore
5	*Canthigaster rostrata*	Sharpnose puffer	Tropical	30	0.30 ± 0.12	0.18 ± 0.06	AR	Omnivore
6	*Mulloidichthys martinicus*	Yellow goatfish	Subtropical	32	0.27 ± 0.26	0.06 ± 0.06	AR	Benthic Carnivore
7	*Bodianus rufus*	Spanish hogfish	Tropical	32	0.21 ± 0.09	0.03 ± 0.02	AR	Invertivore; eat parasites from large fish when juveniles
8	*Anisotremus surinamensis*	Black margate	Subtropical	30	0.17 ± 0.12	0.07 ± 0.05	AR	Invertivore; also zooplankton and small fish
9	*Holacanthus ciliaris*	Queen angelfish	Subtropical	32	0.05 ± 0.03	0.03 ± 0.03	AR	Invertivore
10	*Pareques acuminatus*	High hat	Tropical	32	0.03 ± 0.02	0.02 ± 0.02	AR	Benthic Carnivore

11	*Calamus penna*	Sheepshead porgy	Tropical	30	0.69 ± 0.23	1.28 ± 0.50	NR	Invertivore
12	*Stegastes variabilis*	Cocoa damselfish	Tropical	30	0.12 ± 0.05	0.55 ± 0.34	NR	Herbivore
13	*Sparisoma atomarium*	Green blotch parrotfish	Tropical	32	0.02 ± 0.01	0.06 ± 0.04	NR	Herbivore
14	**Abudefduf taurus*	Night sergeant	Subtropical	24	0.00 ± 0.00	0.01 ± 0.01	NR_ exclusive	Omnivore; also algae
15	*Chromis enchrysura*	Yellowtail reeffish	Tropical	32	0.00 ± 0.00	0.02 ± 0.02	NR_ exclusive	Planktivore
16	*Haemulon album*	White margate	Tropical	33	0.00 ± 0.00	0.01 ± 0.01	NR_ exclusive	Invertivore
17	*Serranus baldwini*	Lantern bass	Tropical	25	0.00 ± 0.00	0.02 ± 0.02	NR_ exclusive	Benthic Carnivore
18	*Serranus phoebe*	Tattler bass	Subtropical	32	0.00 ± 0.00	0.02 ± 0.02	NR_ exclusive	Invertivore
19	*Serranus tigrinus*	Harlequin bass	Tropical	32	0.00 ± 0.00	0.02 ± 0.02	NR_ exclusive	Invertivore

Supplementary References

1. Froese, R. & Pauly, D. FishBase. (2018). at <www.fishbase.org>
2. Whitfield, P. E. *et al.* Native fish community structure and Indo-Pacific lionfish *Pterois volitans* densities along a depth-temperature gradient in Onslow Bay, North Carolina, USA. *Mar. Ecol. Prog. Ser.* **509,** 241–254 (2014).
