# Supplementary material for: Artificial reefs facilitate tropical fish at their range edge
Source: Commun Biol. 2019 May 6;2:168. doi: 10.1038/s42003-019-0398-2 (PMC6502939; doi:10.1038/s42003-019-0398-2)
Supplement: Supplementary file 2 — Reporting Summary [file 42003_2019_398_MOESM2_ESM.pdf]

nature research

Corresponding author(s): Avery Paxton

Reporting Summary

Nature Research wishes to improve the reproducibility of the work that we publish. This form provides structure for consistency and transparency in reporting. For further information on Nature Research policies, see Authors & Referees and the Editorial Policy Checklist.

Statistical parameters

When statistical analyses are reported, confirm that the following items are present in the relevant location (e.g. figure legend, table legend, main text, or Methods section).

n/a	Confirmed	
☐	☒	The exact sample size (*n*) for each experimental group/condition, given as a discrete number and unit of measurement
☐	☒	An indication of whether measurements were taken from distinct samples or whether the same sample was measured repeatedly
☐	☒	The statistical test(s) used AND whether they are one- or two-sided<br>*Only common tests should be described solely by name; describe more complex techniques in the Methods section.*
☐	☒	A description of all covariates tested
☐	☒	A description of any assumptions or corrections, such as tests of normality and adjustment for multiple comparisons
☐	☒	A full description of the statistics including central tendency (e.g. means) or other basic estimates (e.g. regression coefficient) AND variation (e.g. standard deviation) or associated estimates of uncertainty (e.g. confidence intervals)
☐	☒	For null hypothesis testing, the test statistic (e.g. *F*, *t*, *r*) with confidence intervals, effect sizes, degrees of freedom and *P* value noted<br>*Give P values as exact values whenever suitable.*
☒	☐	For Bayesian analysis, information on the choice of priors and Markov chain Monte Carlo settings
☐	☒	For hierarchical and complex designs, identification of the appropriate level for tests and full reporting of outcomes
☐	☒	Estimates of effect sizes (e.g. Cohen's *d*, Pearson's *r*), indicating how they were calculated
☐	☒	Clearly defined error bars<br>*State explicitly what error bars represent (e.g. SD, SE, CI)*

Our web collection on statistics for biologists may be useful.

Software and code

Policy information about availability of computer code

Data collection	Data were collected on diving-conducted surveys, as described in the MS.
Data analysis	Data were analyzed in R, as stated in the MS.

For manuscripts utilizing custom algorithms or software that are central to the research but not yet described in published literature, software must be made available to editors/reviewers upon request. We strongly encourage code deposition in a community repository (e.g. GitHub). See the Nature Research guidelines for submitting code & software for further information.

Data

Policy information about availability of data

All manuscripts must include a data availability statement. This statement should provide the following information, where applicable:

- Accession codes, unique identifiers, or web links for publicly available datasets
- A list of figures that have associated raw data
- A description of any restrictions on data availability

The datasets generated during and/or analyzed during the current study are available from the corresponding author on reasonable request.

Field-specific reporting

Please select the best fit for your research. If you are not sure, read the appropriate sections before making your selection.

☐ Life sciences ☐ Behavioural & social sciences ☒ Ecological, evolutionary & environmental sciences

For a reference copy of the document with all sections, see nature.com/authors/policies/ReportingSummary-flat.pdf

Ecological, evolutionary & environmental sciences study design

All studies must disclose on these points even when the disclosure is negative.

Study description	This is an observational study of reef fishes occupying natural rocky reefs and artificial habitats on the continental shelf of North Carolina. We collected data on fishes, as well as environmental variables, during SCUBA-diver conducted surveys.
Research sample	We conducted SCUBA-diver surveys of thirty temperate reefs off the coast of NC along the southeastern USA continental shelf. Sites were sampled seasonally during 2013 – 2015.
Sampling strategy	To quantify fish abundance, divers sampled along a 30-m x 4-m (120-m2) belt transect 38–40, while recording species and abundance of all fishes present throughout the water column. Each belt-transect included both conspicuous and cryptic categories of reef fishes that were identified to the lowest taxonomic level possible. We collected measurements of the depth and complexity of each reef and the water temperature on each reef using an Onset HOBO U20 Titanium Water Level Logger.<br><br>We used generalized linear models (GLMs) to test whether reef type (artificial, natural), depth, water temperature, and structural complexity affected abundance and biomass of fishes by individual climate ranges. To investigate potential mechanisms of patterns observed in the abundance and biomass models, we constructed several additional models. First, we modeled the relationship between reef type and fish species richness by constructing GLMs with a Poisson distribution for each climate range. Second, to investigate differences in abundance of functional groups by reef type, we constructed GLMs with a negative-binomial error distribution and a log-link function. Third, we used two-sample t-tests to quantify differences in abundance of select fishes by reef type.
Data collection	Data were collected by trained scientific divers, as per above.
Timing and spatial scale	See above.
Data exclusions	Data were not excluded from analyses.
Reproducibility	All analyses are reproducible. Data will be archived in Dryad. Code are maintained by authors and available upon request.
Randomization	This was an observational study so experimental randomization was not conducted.
Blinding	Blinding was not used for this study for data collection because in person observations of fishes were required.

Did the study involve field work? ☒ Yes ☐ No

Field work, collection and transport

Field conditions	Underwater surveys of thirty reefs on the continental shelf of North Carolina, USA
Location	Reefs in Onslow Bay and Long Bay, North Carolina, USA
Access and import/export	N/A - Observational study
Disturbance	N/A - Observational study

Reporting for specific materials, systems and methods

Materials & experimental systems

n/a	Involved in the study
☒	☐ Unique biological materials
☒	☐ Antibodies
☒	☐ Eukaryotic cell lines
☒	☐ Palaeontology
☐	☒ Animals and other organisms
☒	☐ Human research participants

Methods

n/a	Involved in the study
☒	☐ ChIP-seq
☒	☐ Flow cytometry
☒	☐ MRI-based neuroimaging

Animals and other organisms

Policy information about studies involving animals; ARRIVE guidelines recommended for reporting animal research

Laboratory animals	N/A
Wild animals	Ethics approval was not required, as this was an observational study where fishes were visually counted and identified in situ by scientific divers.
Field-collected samples	N/A
